# Editorial: Nutrition in prevention and management of non-alcoholic fatty liver disease

**DOI:** 10.3389/fnut.2023.1212363

**Published:** 2023-07-13

**Authors:** Matina Kouvari, Domenico Sergi, Manja Zec, Nenad Naumovski

**Affiliations:** ^1^Discipline of Nutrition and Dietetics, Faculty of Health, University of Canberra, Canberra, ACT, Australia; ^2^Functional Foods and Nutrition Research (FFNR) Laboratory, University of Canberra, Bruce, Ngunnawal Country, ACT, Australia; ^3^Department of Translational Medicine, University of Ferrara, Ferrara, Italy; ^4^School of Nutritional Sciences and Wellness, University of Arizona, Tucson, AZ, United States

**Keywords:** non-alcoholic fatty liver disease, liver steatosis, nutrition, food pattern, nutrient, bioactive compound, cardiovascular disease, cardiometabolic implications

The overall clinical burden of non-alcoholic fatty liver disease (NAFLD) has increased steadily since the 1980′s affecting 25% of individuals with around three times higher prevalence within population affected by type 2 diabetes mellitus (T2DM) (1, 2). In addition, non-alcoholic steatohepatitis (NASH) has reached epidemic proportions affecting between 1.5% to 6.5% of the general population and as many as 37% of people living with T2DM (1, 2). Furthermore, 20%−30% of patients with NAFLD develop NASH, which can lead to cirrhosis and associated complications, including hepatocellular cancer. Identification and management of liver steatosis and fibrosis that ensue may contribute to the early prevention and treatment of NASH. Furthermore the fibrosis, liver outcomes and cardiovascular disease (CVD), are the main cause of morbidity and mortality among subjects with NASH (3). Additionally, NASH is associated with a substantially increased risk of new-onset T2DM and risk varies markedly from a 33% increase (4) to a 5.5-fold increase in risk (5).

Currently, there are no medical treatment available for the management of NASH with the lifestyle changes remaining the first-line treatment strategy. Emphasis is focused on the weight loss (6) but sustainability of current hypocaloric diets that may be used in the management of NASH is low. Mediterranean diet is regarded as one of the healthiest dietary patterns and it currently represents the “*gold standard”* in preventive medicine, mainly due to the harmonic combination of many foods with antioxidant and anti-inflammatory properties (7). Although several guidelines recommend the Mediterranean diet as a promising pattern for NASH management (6), no dietary pattern (including the Mediterranean diet) has been adequately investigated (8). In a study by Salehi-Sahlabadi et al. specific dietary patterns were revealed through unsupervised analysis and they were associated with the presence of NAFLD. They reported a higher intake of fruit and nuts, high fiber foods and optimal dietary fat profile in concert with low sugar intake may be associated with the lower odds of developing NAFLD. The Mediterranean diet is characterized by food diversity and the use of various herbs and spices in order to enhance meal flavor while reducing the use of salt and other flavor enhancers. These two characteristics were examined in the context of a cross-sectional study in Chinese population against the likelihood of NAFLD diagnosis. This study revealed that salt intake increased the probability of being diagnosed with NAFLD, whereas food diversity acted as a protective factor for NAFLD (Luo et al.).

Questions are raised about which specific macronutrients may mediate the beneficial effects elicited by the Mediterranean diet on NAFLD and cardiometabolic health and the putative molecular mechanisms underpinning these effects (6). In this paradigm, total fat intake was not related to liver steatosis in line with a recent findings (9). Carbohydrates, glycemic load and added sugars are pivotal factors affecting liver health (10, 11). Vitamins and other micronutrients have also been investigated in relation to liver health with contradictive outcomes. In this context, there is an emerging interest in vitamin B12 due to the storage of this vitamin in the liver. Mechanistically the relationship between vitamin B12 and liver health is potentially explained by the implication of this vitamin in the metabolism of homocysteine which, in turn, may have cardiometabolic implications (12). A Mendelian Randomization study suggested that genetically predicted higher Vitamin B12 levels were consistently associated with increased odds of NAFLD even if no association was revealed with liver enzymes (Fu et al.); more studies are needed to confirm these observations in larger global populations.

Sarcopenia is the involuntary reduction of muscle mass and decline in strength. It is also a combination of quantitative (reduction in motor unit number) and qualitative (muscle fiber atrophy) characteristics (13). Similarly, the sarcopenic obesity, is the coexistence of sarcopenia with high fat mass and both conditions in addition to NAFLD are also associated with the development of metabolic syndrome and its components (14). These are also silent, progressive conditions, closely linked with aging and still remain largely undiagnosed. There are limited data linking NAFLD with sarcopenia and sarcopenic obesity (15). In a meta-analysis of cross-sectional studies, patients with NAFLD had lower skeletal muscle mass index, which is an indicator of sarcopenia, than controls (16). Furthermore, patients with sarcopenia had higher rates of NAFLD, NASH and significant hepatic fibrosis compared with those without sarcopenia (17). A large cohort study from the Third National Health and Nutrition Examination Survey (NHANES III) showed that sarcopenia was associated with higher overall mortality in patients with NAFLD, but not those without NAFLD (Luo et al.). A retrospective study in Chinese adults with visceral adiposity investigated the gender- and age-specific association between low muscle mass and the presence of NAFLD (Luo et al.). In this study, SMI was significantly lower in men and middle-aged women with NAFLD than in those without NAFLD. An inverse relationship with the presence of NAFLD in both men and women was also observed; muscle mass was associated with a higher likelihood of NAFLD in middle-aged females and males of any age.

Prevention and management of NAFLD still remains an active scientific field. The recommendations such as adhering to healthier dietary patterns (Mediterranean diet) may provide beneficial health outcomes and should be still considered as the promising management of the NASH. This approach should also include adequate strategies for the prevention of the loss of the muscle mass and decline in strength. More research should be orientated toward determining the paths that result in impaired liver health to drive effective and management solutions in terms of dietary and overall lifestyle interventions.

## Author contributions

MK, DS, MZ, and NN were editors of this Research Topic. MK has produced a first draft. DS, MZ, and NN have provided the input, review, and editing. All authors have read and approved the final version of this Editorial.
